# Supplementary material for: RaptorX-Angle: real-value prediction of protein backbone dihedral angles through a hybrid method of clustering and deep learning
Source: BMC Bioinformatics. 2018 May 8;19(Suppl 4):100. doi: 10.1186/s12859-018-2065-x (PMC5998898; doi:10.1186/s12859-018-2065-x)
Supplement: Supplementary file 1 — Supplementary Material. This additional file contains two parts: S1. supplementary methods for vector representation and normalisation of angles, and also feature generation; S2. supplementary results for determining regularization factor, testing different network architectures, testing different number of labels to use for real value prediction and performance comparison. (PDF 250 kb) [file 12859_2018_2065_MOESM1_ESM.pdf]

Supplementary Material for “RaptorX-Angle: real-value and confidence prediction of protein backbone dihedral angles through a hybrid method of clustering and deep learning”

Yujuan Gao[1,2], Sheng Wang[2], Minghua Deng[1,3,4*], Jinbo Xu[2*]

[1] Center for Quantitative Biology, Peking University, Beijing, China
[2] Toyota Technological Institute at Chicago, Chicago, Illinois, United States of America
[3] School of Mathematical Sciences, Peking University, Beijing, China
[4] Center for Statistical Sciences, Peking University, Beijing, China

S1 Supplemental Methods

S1.1 Vector representation of angles

For a certain angle θ, we can equivalently denote it by a vector $\mathbf{w} = (\cos(\theta), \sin(\theta))$. Reversely, given a vector representation $\mathbf{w} = (w_0, w_1)$ of an angle θ, where $w_0^2 + w_1^2 = 1$, the corresponding angle can be derived from:

$$\theta = \begin{cases} \theta_0 & \text{if } w_0 \geq 0 \\ \theta_0 + \pi & \text{if } w_0 < 0 \text{ and } w_1 > 0 \\ \theta_0 - \pi & \text{else.} \end{cases}$$

where $\theta_0 = \arctan(\frac{w_1}{w_0})$. Similarly, a dihedral angle pair (ϕ, ψ) can be denoted as

$$\mathbf{v} = (\cos(\phi), \sin(\phi), \cos(\psi), \sin(\psi)).$$

And given the vector representation $\mathbf{v} = (v_0, v_1, v_2, v_3)$, where $v_0^2 + v_1^2 = 1$ and $v_2^2 + v_3^2 = 1$, we can easily derive the corresponding angles ϕ and ψ.

*Corresponding authors

S1.2 Normalisation of angle vectors

For each vector $\mathbf{C} = (c_0, c_1, c_2, c_3)$, we did normalisation as follows:

$$\begin{aligned} \widetilde{\mathbf{C}} &= (\widetilde{c}_0, \widetilde{c}_1, \widetilde{c}_2, \widetilde{c}_3) \\ &= (\frac{c_0}{\sqrt{{c_0}^2 + {c_1}^2}}, \frac{c_1}{\sqrt{{c_0}^2 + {c_1}^2}}, \\ &\quad \frac{c_2}{\sqrt{{c_2}^2 + {c_3}^2}}, \frac{c_3}{\sqrt{{c_2}^2 + {c_3}^2}}). \end{aligned}$$

so that each vector $\widetilde{\mathbf{C}}$ is a valid representation for some angle pair.

S1.3 Feature generation

For each site in each sequence protein, we run BuildAli from HHpred [1, 2] with default parameters and 2 iterations to search against UniRef90 to generate position-specific frequency matrix (PSFM) and calculate corresponding scores. Then we run PSI-BLAST [3] with E-value threshold 0.001 to search against nr90 database to generate the position specific scoring matrix(PSSM) and transform PSSM by the sigmoid function $1/(1 + \exp(-x))$, where x is an entry of PSSM matrix. We also use a binary vector of 20 elements to indicate the amino acid type, as well as predicted solvent accessibility (ACC) probabilities from RaptorX [4] and predicted secondary structure(SS) probabilities from PSIPRED [5]. In total there are 66 input features for each residue, in which 20 from PSSM, 20 from PSFM, 20 from primary sequence, 3 from ACC prediction and 3 from SS prediction.

S2 Supplemental Results

S2.1 Determining regularization factor

There is only one hyper-parameter λ, which is used to avoid overfitting, to be tuned. To choose the proper value and test the stability of our model, we conduct a five-fold cross validation. That is, we randomly divide the TR5046 into 5 subsets and use 4 as λ-training set and 1 as λ-validation set. With network architecture fixed ($N_{layers} = 5$, $N_{nodes} = 100$, $halfWinSize = 3$), we train on λ-training sets and calculate the loss on λ-validation sets as the measure of performance.

Actually, our method gains good performance as long as the negative log-likelihood item is about 10-50 times larger than the regularization item. Specifically, the regularization factor λ lies in 0.0008-0.0015 and the performance is rather robust to different λ (Figure S1). Once λ is fixed, we can estimate all the model parameters by solving optimization problems.

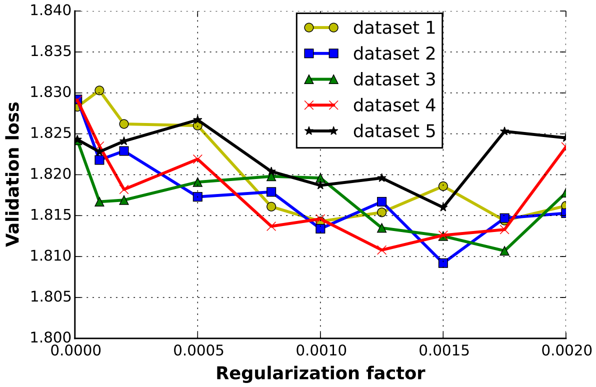

Figure S1: Five-fold cross-validation results of loss on TR5046 with different regularization factors. Specifically, TR5046 is divided equally into 5 subsets, 4 is chosen as λ-training set and 1 as λ-validation set, resulting in 5 datasets corresponding to 5 combinations of training and validation set.

S2.2 Testing different network architectures

The architecture of our deep learning models is mainly determined by 3 factors: (i) the number of hidden layers N_{layers}; (ii) the number of different neuron nodes N_{nodes} at each layer; (iii) the half window size at each layer $halfWinSize$. To choose proper network architectures, we do experiments for each factor and kept the other two fixed on the λ-training sets and λ-validation sets. Figure S2 shows the five-fold cross-validation results of loss with different architectures. From Figure S2(A), the loss on λ-validation set slightly decreases when the half of window size $halfWinSize$ changes to 3 from 1 and rapidly increases when $halfWinSize = 3$ gets larger. So we fix $halfWinSize = 3$. Figure S2(B) indicates that the number of nodes in each layer N_{nodes} does not have any obvious pattern. But when $N_{nodes} = 100$, it gets smallest loss almost on each set. So we fix $N_{nodes} = 100$. As is shown in Figure S2(C), with increasing network depth N_{layers}, loss decreases first and increases consistently when $N_{layers} > 30$. The loss when $N_{layers} > 50$ would be larger than it when $N_{layers} = 1$ or 3. In consideration of model representation ability and computational difficulty trade-off, we use the mean of an ensemble of 6 networks with common $N_{nodes} = 100$, $halfWinSize = 3$ and different $N_{layers} = 5, 10, 20, 30, 40, 50$.

S2.3 Testing different number of labels to use for real-value angle prediction

Firstly, we test Mean Absolute Error (MAE) performance using gold standard label, i.e., take corresponding cluster centre (ϕ, ψ) pair as the final real-value angle prediction and calculate the corresponding MAE to see the theoretical

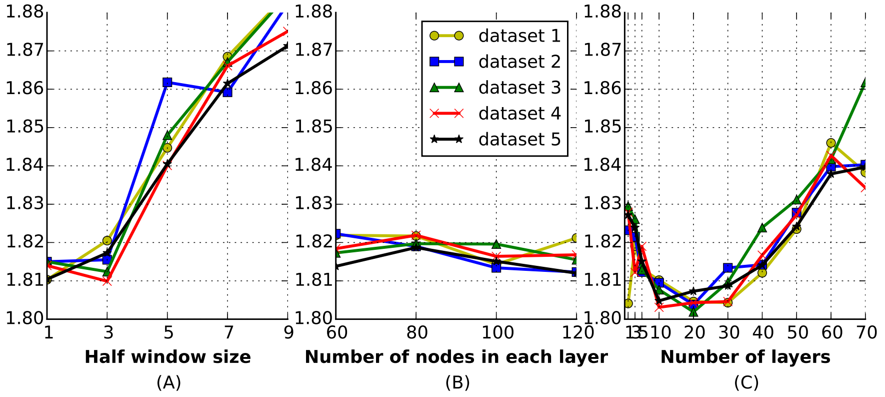

Figure S2: Five-fold cross-validation results of validation loss on TR5046 with different network architectures. (A)Loss results for networks with different half window size and common $N_{layers} = 5$, $N_{nodes} = 100$; (B)Loss results for networks with different number of neurons at each layer and common $N_{layers} = 5$, $halfWinSize = 3$; (C)Loss results for networks with different number of hidden layers and common $halfWinSize = 3$, $N_{nodes} = 100$.

limit using single labels to predict real-value angles. Then we test different number of labels with top probabilities. Table S1 shows the MAE result using different choice of labels to derive real-value angle predictions. It is surprising that we could do very well with single true labels, which may indicate the clusters are well enough to reflect the angle information. However, it is hard to train a perfect classifier. To balance the bias introduced from the classifier, we test R labels with top probabilities. The MAE performance improves rapidly when R increases to 10, and steadily after. Overall, using all labels has gained the best performance, which we have adopted in following studies.

Table S1: The Mean Absolute Error for real-value predictions with different number of labels on TS1267.

(°)	Phi	Psi	Phi_H	Psi_H	Phi_E	Psi_E	Phi_C	Psi_C
Gold	6.76	6.98	4.41	4.15	7.79	8.11	8.53	9.19
Top-1	20.10	28.31	8.98	13.01	21.00	23.95	30.88	46.53
Top-5	18.61	27.77	8.71	12.85	19.38	22.65	29.12	44.74
Top-10	18.35	26.86	8.64	**12.63**	18.28	21.06	28.89	44.31
Top-15	18.10	26.69	8.43	12.92	18.26	20.95	27.96	44.12
Top-20	**18.08**	**26.68**	**8.35**	12.98	**18.24**	**20.94**	**27.88**	**44.11**

Gold: prediction with gold standard label.
Top-R: prediction with top R probable labels.

Table S2: Pearson correlation coefficient of sine values between predicted and true angles .

	**TS1267**	**CASP11**	**CASP12**
	$\sin(\phi)/\sin(\psi)$	$\sin(\phi)/\sin(\psi)$	$\sin(\phi)/\sin(\psi)$
RaptorX-Angle	0.6934/0.7891	0.6257/0.7443	0.6270/0.7107
SPIDER2	0.6904/0.7719	0.6256/0.7376	0.6142/0.6952
SPINE X	0.6316/0.7208	0.5195/0.5544	0.5096/0.5656
ANGLOR	0.5484/0.6726	0.4613/0.6364	0.4744/0.6081

S2.4 Overall PCC performance of sine values compared with other methods

Table S2 shows the Pearson Correlation Coefficient (PCC) performance of sine values on the three benchmarks. RaptorX-Angle has gained the highest PCC on all datasets. The advantage is more obvious on TS1267 and CASP12.

S2.5 Overall two-state accuracy performance compared with other methods

As angles reflect the backbone conformation, angle values are variable due to the protein backbone flexibility. So reducing large angle errors is important for conformation sampling. As the distributions of ϕ and ψ both have two peaks, they can be divided into two states related to their peaks. Here we adopt the same two-state definitions with [6]. That is, $[0°, 150°]$ and the rest for ϕ; $[-100°, 60°]$ and the rest for ψ. We calculated two-state prediction accuracy to see if there is any improvement in large angle errors. Table S3 show the results on TS1267 dataset, 85 CASP11 targets and 40 CASP12 targets. RaptorX-Angle performs the best on TS1267, regardless of secondary structure types, and has about 0.15 and almost 1 percent improvement over SPIDER2 for ϕ and ψ, respectively. On CASP11 dataset, RaptorX-Angle gains comparable performance with SPIDER2. On the CASP12 targets, RaptorX-Angle has 0.2 and 0.91 percent improvement over the best SPIDER2 among other methods for ϕ and ψ, respectively.

S2.6 Supplementary result for the relationship between prediction error and standard deviation

To demonstrate representation power of the eight points, which stand for the mean for different secondary structural regions, we get the scatter plot of the whole cloud of all the clusters (see Figure S3). There are 120 markers in Figure S3 for 20 clusters. Each cluster has been divided into 6 subsets indicating three kinds of secondary structural regions, i.e., helix (red), strand (greed) and coil (blue), and two kinds of dihedral angles, i.e., ϕ (circle) and ψ (triangle). Each marker demonstrates the mean of the corresponding subset in each cluster

Table S3: Two-state accuracy of four methods for different regions on three benchmarks: TS1267, 72 CASP11 targets and 40 CASP12 targets.

(%)	Phi	Psi	Phi_H	Psi_H	Phi_E	Psi_E	Phi_C	Psi_C
**TS1267**								
RaptorX-Angle	96.84	88.70	99.29	96.15	98.77	94.08	93.19	78.88
SPIDER2	96.71	87.87	99.28	95.37	98.65	92.92	92.96	77.33
SPINE X	96.28	85.10	99.12	94.38	98.24	87.92	92.27	74.09
ANGLOR	95.48	82.33	99.23	93.85	98.31	86.64	90.04	68.18
**CASP11**								
RaptorX-Angle	96.16	86.56	99.02	94.81	98.76	92.35	91.90	76.16
SPIDER2	96.13	86.55	98.96	94.47	98.49	92.16	92.12	76.40
SPINE X	94.87	77.48	98.16	86.87	97.46	79.94	90.28	67.63
ANGLOR	94.98	80.62	99.05	93.84	98.38	83.75	89.19	66.99
**CASP12**								
RaptorX-Angle	95.84	85.66	99.41	94.70	98.37	90.80	91.20	74.58
SPIDER2	95.81	84.67	99.51	94.26	98.29	89.51	91.12	73.36
SPINE X	94.73	77.65	99.07	89.12	97.69	78.60	89.18	66.75
ANGLOR	94.58	80.13	99.41	92.25	97.99	83.90	88.36	67.10

Phi and Psi denote MAE for all residues;
Phi_H and Psi_H denote MAE for residues in helix region;
Phi_E and Psi_E denote MAE for residues in beta strand region;
Phi_C and Psi_C denote MAE for residues in coil region.

with the marker size scales with the subset size. The bold black line is the fitted line from the 8 points. We could see that the markers with medium or big size distribute rather evenly on both sides of the fitted line. So it is fair enough to use the 8 points to fit the linear model.

References

[1] Söding, J.: Protein homology detection by hmm–hmm comparison. Bioinformatics **21**(7), 951–960 (2004)

[2] Remmert, M., Biegert, A., Hauser, A., Söding, J.: Hhblits: lightning-fast iterative protein sequence searching by hmm-hmm alignment. Nature methods **9**(2), 173–175 (2012)

[3] Camacho, C., Coulouris, G., Avagyan, V., Ma, N., Papadopoulos, J., Bealer, K., Madden, T.L.: Blast+: architecture and applications. BMC bioinformatics **10**(1), 421 (2009)

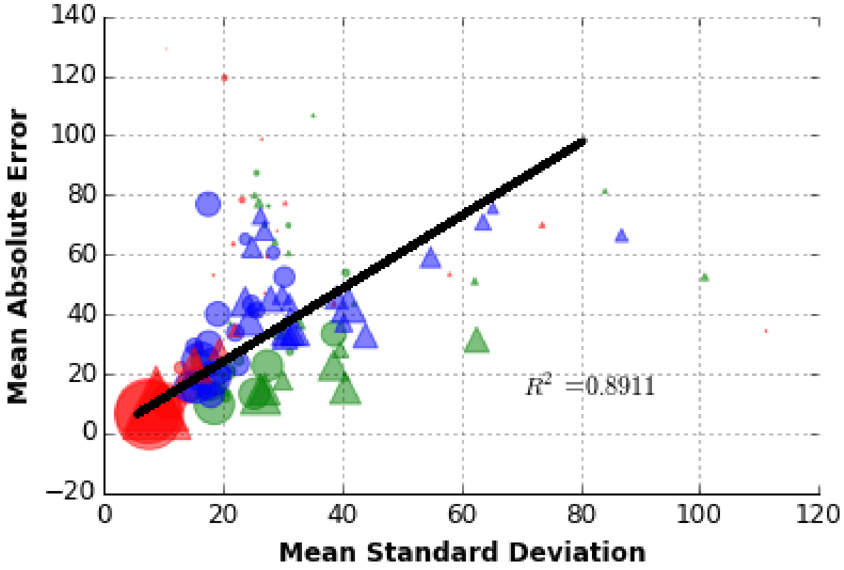

Figure S3: Relationship between prediction error and standard deviation. The bold black line is the fitted line from the 8 points. There are also 120 markers for 20 clusters. Each cluster has been divided into 6 subsets indicating three kinds of secondary structural regions, i.e., helix (red), strand (greed) and coil (blue), and two kinds of dihedral angles, i.e., ϕ (circle) and ψ (triangle). Each marker demonstrates the mean of the corresponding subset in each cluster with the marker size scales with the subset size.

[4] Wang, S., Li, W., Liu, S., Xu, J.: Raptorx-property: a web server for protein structure property prediction. Nucleic acids research **44**(W1), 430–435 (2016)

[5] Jones, D.T.: Protein secondary structure prediction based on position-specific scoring matrices. Journal of molecular biology **292**(2), 195–202 (1999)

[6] Heffernan, R., Paliwal, K., Lyons, J., Dehzangi, A., Sharma, A., Wang, J., Sattar, A., Yang, Y., Zhou, Y.: Improving prediction of secondary structure, local backbone angles, and solvent accessible surface area of proteins by iterative deep learning. Scientific reports **5** (2015)
